# AQP4-independent TRPV4 modulation of plasma membrane water permeability

**DOI:** 10.3389/fncel.2023.1247761

**Published:** 2023-08-31

**Authors:** Barbara Barile, Maria Grazia Mola, Francesco Formaggio, Emanuela Saracino, Antonio Cibelli, Concetta Domenica Gargano, Guido Mogni, Antonio Frigeri, Marco Caprini, Valentina Benfenati, Grazia Paola Nicchia

**Affiliations:** ^1^Department of Bioscience, Biotechnology and Environment, University of Bari Aldo Moro, Bari, Italy; ^2^Department of Pharmacy and Biotechnology, University of Bologna, Bologna, Italy; ^3^Institute for Organic Synthesis and Photoreactivity (ISOF), National Research Council of Italy (CNR), Bologna, Italy; ^4^Department of Translational Biomedicine and Neuroscience (DiBraiN), School of Medicine, University of Bari Aldo Moro, Bari, Italy; ^5^Dominick P. Purpura Department of Neuroscience, Albert Einstein College of Medicine, 840 Kennedy Center, Bronx, NY, United States

**Keywords:** water channels, ion channels, TRPV4, AQP4, calcium ions, cell swelling, astrocytes

## Abstract

Despite of the major role of aquaporin (AQP) water channels in controlling transmembrane water fluxes, alternative ways for modulating water permeation have been proposed. In the Central Nervous System (CNS), Aquaporin-4 (AQP4) is reported to be functionally coupled with the calcium-channel Transient-Receptor Potential Vanilloid member-4 (TRPV4), which is controversially involved in cell volume regulation mechanisms and water transport dynamics. The present work aims to investigate the selective role of TRPV4 in regulating plasma membrane water permeability in an AQP4-independent way. Fluorescence-quenching water transport experiments in *Aqp4*^–/–^ astrocytes revealed that cell swelling rate is significantly increased upon TRPV4 activation and in the absence of AQP4. The biophysical properties of TRPV4-dependent water transport were therefore assessed using the HEK-293 cell model. Calcein quenching experiments showed that chemical and thermal activation of TRPV4 overexpressed in HEK-293 cells leads to faster swelling kinetics. Stopped-flow light scattering water transport assay was used to measure the osmotic permeability coefficient (*Pf*, cm/s) and activation energy (*Ea*, kcal/mol) conferred by TRPV4. Results provided evidence that although the *Pf* measured upon TRPV4 activation is lower than the one obtained in AQP4-overexpressing cells (*Pf* of AQP4 = 0.01667 ± 0.0007; *Pf* of TRPV4 = 0.002261 ± 0.0004; *Pf* of TRPV4 + 4αPDD = 0.007985 ± 0.0006; *Pf* of WT = 0.002249 ± 0.0002), along with activation energy values (*Ea* of AQP4 = 0.86 ± 0.0006; *Ea* of TRPV4 + 4αPDD = 2.73 ± 1.9; *Ea* of WT = 8.532 ± 0.4), these parameters were compatible with a facilitated pathway for water movement rather than simple diffusion. The possibility to tune plasma membrane water permeability more finely through TRPV4 might represent a protective mechanism in cells constantly facing severe osmotic challenges to avoid the potential deleterious effects of the rapid cell swelling occurring via AQP channels.

## Graphical Abstract

Model for the intracellular calcium modulation of plasma membrane water permeability in cells under hypotonic conditions. **(A)** Slow lipid-mediated permeation of water molecules through cellular plasma membrane (lowest *Pf* and highest *Ea* values). **(B)** Rapid water transport following intracellular calcium increase upon TRPV4 activation or ER-store depletion in the presence of thapsigargin. Calcium might putatively act on 1. intrinsic biophysical properties of the lipid bilayer (i.e., fluidity; thickness; viscosity) or 2. calcium-dependent transmembrane proteins able to co-transport water molecules together with solutes (intermediate values of *Pf* and *Ea*). **(C)** Fast aquaporin-mediated water entry (highest *Pf* and lowest *Ea* values). 
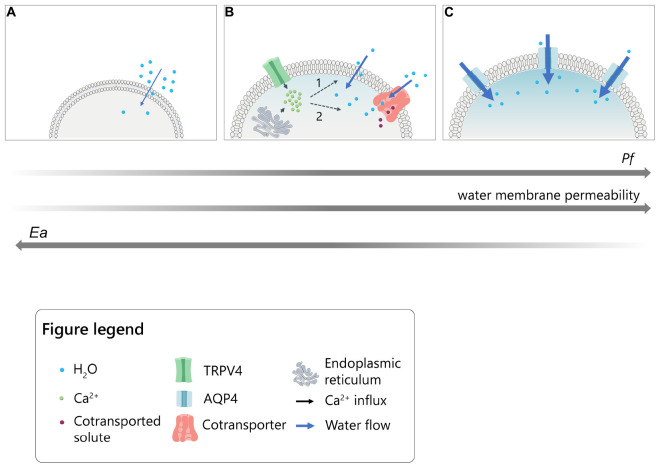

## 1. Introduction

Cells physiologically undergo constant volume fluctuations, in the form of cell swelling or cell shrinking, depending on whether they occur under hypoosmotic or hyperosmotic conditions, respectively (Feher, 2012; Dmitriev et al., 2019). Although this process is primarily due to the simple diffusion of water molecules across plasma membranes, the speed of cell swelling and shrinkage is significantly increased by the expression of the water channels aquaporins (AQPs) that facilitate osmotic water flows, bidirectionally (Agre et al., 1998; Jin et al., 2011). Considering the osmotic water permeability coefficient (*Pf*) as an accurate parameter to define water transport properties of a defined barrier, the expression of AQPs significantly increases water membrane permeability of a cell (*Pf* > 0.01 cm/s) compared to simple diffusion of water molecules (*Pf* < 0.005 cm/s) (Verkman, 2000). The expression of aquaporins is well known to be associated with an increased membrane osmotic water permeability (Verkman, 2013). However, cotransporters have been identified as water-transporting proteins that could provide additional ways for water molecules’ permeation and independently of the osmotic gradient (MacAulay, 2021). In light of this, it is not surprising that different cells were found to exhibit an unexpectedly high water permeability in the absence of AQPs (Jo et al., 2015; Ríos et al., 2019; MacAulay, 2021). In the Central Nervous System (CNS), Aquaporin-4 (AQP4) has been shown to establish a complex with the calcium-channel Transient-Receptor Potential Vanilloid member-4 (TRPV4) and, more specifically, in astrocytes (Benfenati et al., 2011; Jo et al., 2015; Iuso and Križaj, 2016; Sucha et al., 2022). Although TRPV4 role in cell volume regulation mechanisms and swelling is debated, its activation is reported to be associated with enhanced water transport even in the absence of AQP4 in astrocytes (MacAulay, 2021) and retinal Müller cells (Jo et al., 2015), while its inhibition was found to prevent increased water diffusion in the retina of diabetic mice (Ríos et al., 2019).

Based on such evidence and besides its well-characterized involvement in swelling-induced regulatory volume decrease (RVD) mechanisms (Becker et al., 2005; Benfenati et al., 2011; White et al., 2016), an unconventional role of TRPV4 in the modulation of cellular swelling has emerged. However, to what extent and through which mechanisms TRPV4 contributes *per se* to cellular swelling or water membrane permeability has been poorly investigated. The present study aims to dissect the biophysical role of TRPV4 in the homeostatic mechanism of cellular swelling in an AQP4-independent system, providing evidence that AQPs-alternative pathways for water permeation exist and are likely to be tuned in a calcium-dependent manner.

## 2. Materials and methods

**Animals**. WT and *Aqp4*^–/–^ pups with a CD1 genetic background (Basco et al., 2013) were used for dissection in primary astrocyte cultures preparation. All the procedures involving animals were performed accordingly to the European and Italian laws on animal use for research and animal care. This project has been approved by the Italian Health Department (Approved Project no. n°710/2017-PR). Mice were bred in the approved facility at the University of Bari under a 12 h dark-to-light cycle at constant room temperature (23–25°C) and humidity (22 ± 2°C, 75%). The design of experiments was aimed at minimizing the number of animals used and included all the procedures to reduce the animal suffering.

**Astrocytes Primary Cultures**. Mouse astrocyte primary cultures were prepared from newborn pups (P1–P3) as previously described (Nicchia et al., 2000). Cells were cultured in DMEM High Glucose medium supplemented with 10% fetal bovine serum (FBS), 100 U/mL penicillin, and 100 mg/mL streptomycin and maintained at 37°C in a 5% CO_2_ incubator. Cell culture products were purchased from Euroclone.^1^

**Cell Cultures and Transfection**. The human embryonic kidney HEK-293 cell line^2^ was grown in DMEM High Glucose medium supplemented with 10% heat-inactivated fetal bovine serum (FBS), 100 U/ml penicillin and 100 mg/ml streptomycin and maintained at 37°C in a 5% CO_2_ incubator (Pisani et al., 2013). To generate HEK-293 cells stably transfected with AQP4 and TRPV4, pmCherry-N1 human M23-AQP4 and pEGFP-N1 human TRPV4 constructs were used. Stable transfection was carried out using Optifect^3^ as suggested by the manufacturer.

**Short-Interference RNA (siRNA)**. HEK-293 and mouse astrocytes were seeded the day before transfection in complete medium. Transient transfection of siRNAs was carried out using Lipofectamine™ RNAiMAX Transfection Reagent, for HEK-293 cells, and Oligofectamine™ Transfection Reagent, for astrocytes, accordingly to the instructions provided from the manufacturer (Thermo Fisher). The transfection was carried out in Opti-MEM™ medium (Thermo Fisher). Specific silencing was assessed by Western blot experiments. TRPV4 siRNA (Benfenati et al., 2011) was used to silence the expression of the proteins. Scramble siRNA (CGAUGGAGAAGGCCAACUAGGGACU) was used as Ctrl siRNA [Dharmacon Research, Inc. (Lafayette, CO, USA)].

**Antibodies**. The following primary antibodies were used for Western Blot and immunofluorescence: mouse anti-GFAP (G3893 Sigma Aldrich, diluted 1:500), rabbit anti-AQP4 (sc-20812 Santa Cruz Biotechnology, diluted 1:500); rabbit anti-TRPV4 (SAB2104243 Sigma Aldrich, diluted 1:200); mouse anti-AQP1 (sc-25287 Santa Cruz Biotechnology, diluted 1:200); rabbit anti-AQP2 (affinity-purified, diluted 1:3000); mouse anti-AQP3 (sc-518001 Santa Cruz Biotechnology, 1:500); rabbit anti-AQP4 (sc-20812 Santa Cruz Biotechnology, diluted 1:500); rabbit anti-AQP5 (PA536529 Thermo Fisher, diluted 1:300); rabbit anti-AQP6 (PA5103615 Thermo Fisher, diluted 1:500); mouse anti-AQP7 (sc-376407 Santa Cruz Biotechnology, diluted 1:500); mouse anti-AQP8 (sc-81870 Santa Cruz Biotechnology, diluted 1:500); mouse anti-AQP9 (sc-74409 Santa Cruz Biotechnology, diluted 1:500). The following secondary antibodies were used: Horseradish Peroxidase (HRP) conjugated donkey anti-rabbit and anti-mouse antibody (diluted 1:5000) for Western blot and donkey anti-rabbit IgG-Alexa Fluor 594 (diluted 1:1000) for immunofluorescence.

**Immunofluorescence**. Cells were fixed in 4% paraformaldehyde, thoroughly washed in phosphate-buffered saline (PBS) with calcium and magnesium, and permeabilized with 0.3% Triton X-100 in PBS. 1% BSA in PBS was used as blocking agent for 30 min. Cells were then incubated with primary antibodies for 1 h at RT, rinsed, in PBS incubated at RT with Alexa-conjugated secondary antibodies for 1 h and washed. Coverslips were mounted with a mounting medium added with DAPI (50% Glycerol, 0.1% N-Propil-Gallate, 1 μg/ml DAPI in PBS) and observed by epifluorescence microscopy (Leica DM6000). Adobe Photoshop auto contrast tool was used to enhance tonal and color correction of images.

**Western Blot Analysis**. Protein samples were obtained as previously described (Mola et al., 2021), separated by 10% (for TRPV4 detection) and 13% (for AQPs detection) Tris-Glycine-SDS-PAGE and transferred to polyvinylidene fluoride (PVDF) membranes.^4^ Membranes with blotted proteins were incubated with primary antibodies, washed, and incubated with peroxidase-conjugated secondary antibodies. Target proteins were revealed with an enhanced chemiluminescent detection system (see text footnote 3) and visualized on a Chemidoc imaging system.^5^ Densitometric analysis was performed with Image Lab software by using Coomassie-blue staining of total protein as normalization channel.

**Fluorescence-quenching water transport assay**. Cells were seeded on black, clear-bottom 96-well-plates (Corning, NY, USA) at a density of 12,000 cells per well, as previously described (Mola et al., 2009). Cells were loaded with 10 μM Calcein-AM at 37°C for 20 min in growth medium and then rinsed in isotonic PBS. Cytosolic calcein fluorophore exhibits concentration-dependent quenching by intracellular components (proteins or salts) so that measured changes in fluorescence are directly proportional to changes in cell volume. Therefore, as cells expand in response to reduced extracellular osmolarity, the dilution of cytosolic protein concentration by water influx negatively affects the calcein quenching and leads to increased fluorescence signal recorded (Solenov et al., 2004). Fluorescence signal changes were recorded on a Flex Station3 plate reader equipped with an integrated automatic liquid handling module,^6^ able to transfer compounds from a source plate to the assay plate during data acquisition. The hypotonic stimulus was induced by adding an appropriate volume of water to obtain 240 mOsm/L final extracellular osmolarity (60 mOsm/L osmotic gradient). The fluorescence signal increases following the hypotonic stimulus due to cell swelling and then decreases during the regulatory volume decrease (RVD), which tends to restore the isosmotic condition. Experiments were conducted in Dulbecco’s Phosphate Buffered Saline (DPBS). Two different external isotonic solutions were prepared: (a) DPBS added with calcium chelator 1 mM EGTA (used as “calcium-free” DPBS), with no calcium; and (b) DPBS added with 1 mM CaCl_2_ (used as “calcium-containing” DPBS).

**FIGURE 1:**
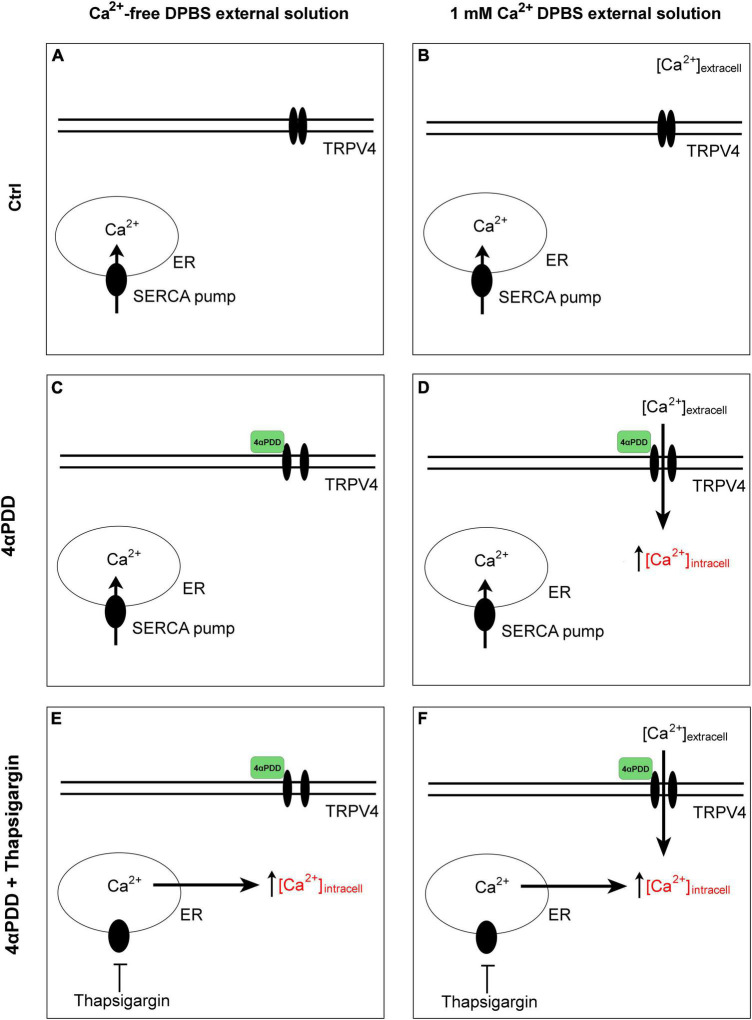
Graphical summary of the design protocols. Control condition in the absence **(A)** and in the presence **(B)** of calcium in the external solution. No intracellular calcium rises are promoted. Activation of TRPV4 by chemical agonist 4αPDD in external medium without **(C)** and with external calcium **(D)**. Activation of TRPV4 by chemical agonist 4αPDD in external medium without **(E)** and with **(F)** external calcium upon thapsigargin stimulation.

Time course fluorescence was recorded at 20°C or at 37°C. Data acquisition was performed using SoftMaxPro software and the data were analyzed with Prism (Graph Pad 8) software. The time constant τ (s) of cell volume variation upon swelling was obtained by fitting the data with an exponential function (One-phase association equation). Calcein fluorescence of representative kinetics is expressed in Arbitrary Units (A.U.) of fluorescence intensity ratios (F/F0), where F0 represents the background. The arrows indicate the time at which hypotonic shock was induced. The graphical schematic in Figure 1 summarizes the experimental conditions used.

**Intracellular calcium measurements**. 24 h after plating in 96-well black-walled, clear bottom plates, cells were loaded with 8 μM Fura-2-AM in DMEM growth medium for 30 min at 37°C. Cells were then stabilized in the same buffer for 10 min at 37°C. Rapid changes in intracellular calcium levels in response to extracellular stimuli were monitored using the FlexStation3 plate reader set at 37°C. The hypotonic stimulus was induced by adding an appropriate volume of water to obtain 240 mOsm/L final extracellular osmolarity (60 mOsm/L osmotic gradient). Compounds were added from a 96-well reservoir plate with pipette heights, fluid transfer volume and rate of addition optimized to minimize disturbance of the cells while ensuring rapid mixing. The fluorescence ratio F340/F380 was used as an indicator of cytosolic calcium changes and the maximum intensity of F340/F380 ratio reached after the hypotonic shock or modulators addition as the peak of calcium response. Data analysis was performed using SoftMaxPro and Prism (Graph Pad 8).

**Chemicals**. *Thapsigargin treatment*: Cells were exposed for 3 min to thapsigargin (Sigma), an inhibitor of the sarco-endoplasmic reticulum Ca^2+^ ATPase (SERCA), preliminarily to the hypotonic shock to induce an intracellular calcium rise. Thapsigargin was used at a final concentration of 1 μM. *4α-Phorbol 12,13-didecanoate treatment*: cells were exposed to 4α-Phorbol 12,13-didecanoate solid (4αPDD, Sigma) for 3 min before the induction of the hypotonic shock. 4αPDD was used at a final concentration of 10 μM to assess the contribution of the TRPV4 ion channel to the swelling-induced intracellular calcium increase and rapid cell volume changes. *RN-1734 treatment*: cells were exposed for 3 min to RN-1734 (Sigma) and used at a final concentration of 50 μM.

**Stopped Flow Light Scattering**. Water permeability measurements were carried out using a light scattering method with a stopped-flow SFM-20 instrument (Biologic, Science Instruments, Claix, France). The instrument dead time was 1.6 ms. A circulating water bath was used to set the sample temperature at 10, 20, or 37°C. Cells from 100-mm diameter plastic dishes were incubated in Ca^2+^/Mg^2+^-free PBS containing 50 mM EDTA for 2 min at 37°C. Collected cells were washed two times in PBS and resuspended at a concentration of 2 × 10^6^ cells/ml. A total of 0.1 ml of the cell suspension was mixed rapidly (<1 ms) with an equal volume of hyposmolar PBS to give a 100 mOsm/L inwardly directed osmotic gradient. Immediately after applying the hypotonic shock, water inflow occurs and the cells swell, causing a decrease in scattered light scattering. The kinetics of increasing cell volume were measured from the time course of 90° scattered light intensity changes at 530 nm, for each temperature. Curves were fitted to single exponential curves using Biokine 32 software to compute the rate constant (k).

**Osmotic permeability coefficient (*Pf*) and activation energy (*Ea*) measurements**. The *Pf* (cm/s) was determined according to the following equation:


(1) $$P\,f=\frac{\text{k}}{{\text{V}}_{\text{w}}\times \left(\frac{\text{S}}{\text{V}}\right)\times \Delta \,\mathrm{osm}}$$

where V_w_ is the partial molar volume of water (18 cm^3^/mol), S/V is the ratio of the cell surface area to the initial volume, and Δosm is the applied osmotic gradient. To obtain the S/V ratio, cells were detached with trypsin-EDTA, thoroughly resuspended, and moved to a dish. A few fields with dozens of isolated spheroid cells were imaged while still in suspension at 10X magnification under a Nikon Eclipse Ti inverted optical microscope. Assuming that detached cells can be geometrically considered to be spheres, the average area of cells measured in ImageJ software (191.3 μm^2^ ± 9.27 SEM) was then used to deduce the average volume and then the surface-to-volume ratio (S/V = 0.768). The Arrhenius activation energy (*Ea*, kcal/mol) is defined by the equation ln *Pf* = *Ea*/RT where R is the gas constant and T is absolute temperature. *Ea* was determined from the slope of an Arrhenius plot of ln *Pf* vs. 1/RT. *k*-values obtained from 8 to 11 kinetics from 3 independent experiments were used to calculate *Pf* and *Ea*.

**Statistical analysis.** All data were previously assessed for normality using the Shapiro-Wilk test (alpha = 0.05). When comparing more than three groups, for normally distributed data we have used: ordinary One-Way ANOVA corrected with Newman-Keuls multiple comparison test when equal SDs; Brown-Forsythe and Welch ANOVA corrected with Tamhane’s T2 multiple comparisons test when non-equal SDs. For non-normally distributed data, we have used Kruskal-Wallis corrected for Dunn’s multiple comparison test. When comparing less than three groups, for normally distributed data we have used: unpaired *t*-test when equal SDs; unpaired *t*-test with Welch’s correction when non-equal SDs. For non-normally distributed data, we have used Mann-Whitney test. Data are shown as mean ± SEM and the level of significance was set at *p* < 0.05. When statistical analysis was performed at multiple levels in the same graph, asterisks were used to indicate significant differences between conditions within each cell line, while different letters were used to indicate significant differences between different cell lines under the same conditions (e.g., in *italics*: comparison between controls; in **bold:** comparison between activated cells). Statistical analyses were performed using GraphPad Prism 8 software.

## 3. Results

### 3.1. TRPV4 activation is associated with an increase in astrocyte swelling rates

**FIGURE 2:**
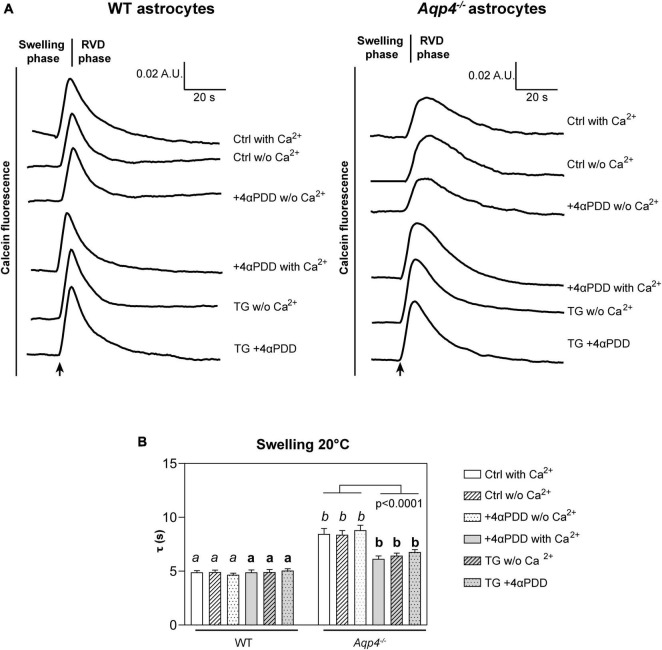
Water transport assay in *Aqp4*^–/–^ and WT astrocyte primary cultures. **(A)** Representative time courses of calcein-AM loaded astrocytes upon exposure to 60 mOsm/L hypotonic gradient at 20°C in the presence or absence of external calcium with 4αPDD and/or thapsigargin (TG) incubation. **(B)** Bar graph showing the time constants (τ) of cell swelling measured in the conditions reported in **(A)**. Data are reported as mean ± SEM and representative of three independent experiments (Table 1). Different letters were used to indicate significant differences between different cell lines under the same conditions (in italics: comparison between controls; in bold: comparison between activated cells). One-way ANOVA with Newman-Keuls multiple comparison tests for WT (ns, *p* > 0.05) and Kruskal-Wallis with Dunn’s multiple comparison test for *Aqp4*^–/–^ astrocytes for comparisons between conditions within each genotype; Kruskal-Wallis with Dunn’s multiple comparison test (in *italics*, *p* < 0.0001) and One-way ANOVA with Newman-Keuls multiple comparisons test (in bold, *p* < 0.0001) for comparison between genotypes under control and activating conditions, respectively. Detailed statistically significant differences are shown in Supplementary Tables 3–5. (For *Aqp4*^–/–:^ Ctrl with Ca^2+^ *n* = 10; Ctrl w/o Ca^2+^ *n* = 10; 4αPDD with Ca^2+^ *n* = 12; 4αPDD w/o Ca^2+^ *n* = 11; TG + 4αPDD *n* = 16; TG w/o Ca^2+^ *n* = 10. For WT: Ctrl with Ca^2+^ *n* = 12; Ctrl w/o Ca^2+^ *n* = 11; 4αPDD with Ca^2+^ *n* = 9; 4αPDD w/o Ca^2+^ *n* = 9; TG + 4αPDD *n* = 9; TG w/o Ca^2+^ *n* = 9).

To evaluate TRPV4 contribution to cell swelling, primary astrocyte cultures endogenously expressing functional TRPV4 and AQP4 were used (Benfenati et al., 2011). In particular, to assess TRPV4 selective role in cell swelling in the presence and the absence of AQP4, water transport measurements were performed in WT and *Aqp4*^–/–^ brain astrocytes. We hence investigated (a) whether TRPV4 activation affects water transport rates and (b) if this phenomenon is specifically associated with TRPV4 activation or with a more general increase in cytosolic calcium, independently of the source. To address these questions, experiments were performed upon TRPV4 activation induced by the chemical activator 4-alpha-Phorbol-12,-13-Didecanoate (4αPDD) promoting calcium influx from the extracellular space. Thapsigargin incubation, either in combination or independently of TRPV4 activation, was also used to promote calcium release from endoplasmic reticulum (ER) stores (Figure 2).

**TABLE 1:** Time constants (τ) of the swelling phase of WT and *Aqp4*^–/–^ astrocytes.

τ (swelling)	WT	*Aqp4* ^–/–^
Ctrl w/o extracellular calcium	4.923 s ± 0.1892	8.376 s ± 0.4060
Ctrl with extracellular calcium	4.893 s ± 0.1675	8.460 s ± 0.5041
+4αPDD with extracellular calcium	4.894 s ± 0.2308	6.149 s ± 0.2712
+4αPDD w/o extracellular calcium	4.665 s ± 0.1532	8.807 s ± 0.4574
Thapsigargin + EGTA	4.921 s ± 0.2288	6.440 s ± 0.2414
Thapsigargin + 4αPDD with extracellular calcium	5.058 s ± 0.1693	6.785 s ± 0.2217

Data are shown as mean ± SEM.

The analysis of WT astrocytes swelling rates revealed no changes in the speed of water transport under all the experimental conditions and regardless of TRPV4 or intracellular calcium modulation, likely due to the presence of AQP4 as major route for water flux. *Aqp4*^–/–^ astrocytes showed slower swelling kinetics compared to WT, in line with previous studies (Solenov et al., 2004). However, in *Aqp4*^–/–^ astrocytes TRPV4 activation in the presence of external calcium was found to be associated with significantly faster water transport rates compared to control conditions. Of note, we found that the cell swelling rate in *Aqp4*^–/–^ astrocytes is enhanced to the same extent under: (a) 4αPDD in the presence of external calcium; (b) thapsigargin in the absence of external calcium; and (c) thapsigargin and 4αPDD in the presence of external calcium (Figure 2B). This indicates that the observed increase in water transport rate is likely mediated by an intracellular calcium increase.

**FIGURE 3:**
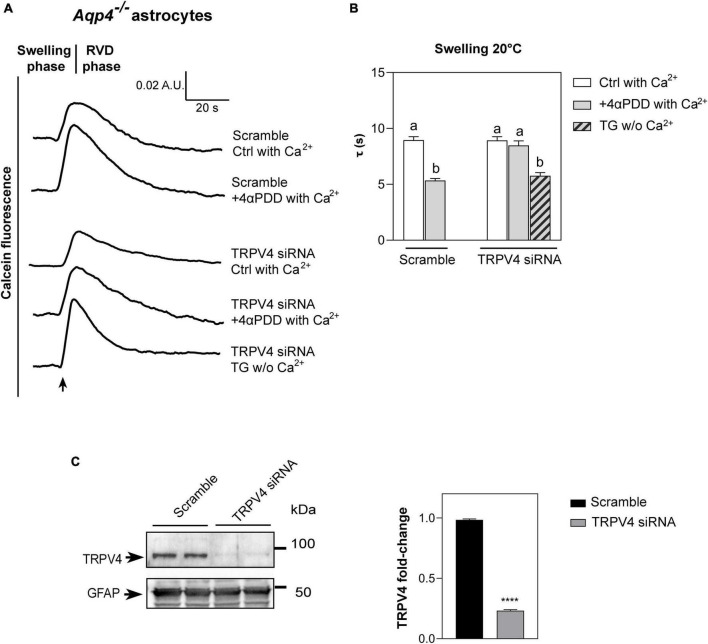
Water transport assay in *Aqp4*^–/–^ astrocytes transfected with TRPV4-siRNA. **(A)** Representative time courses of calcein-AM loaded astrocytes knockdown for TRPV4 upon exposure to 60 mOsm/L hypotonic gradient at 20°C in the presence or absence of external calcium with 4αPDD and/or thapsigargin (TG) incubation. **(B)** Bar graph showing the time constants (τ) of cell swelling measured in the conditions reported in **(A)**. Data are reported as mean ± SEM (Table 2). Kruskal-Wallis with Dunn’s multiple comparison test for comparison between conditions. Detailed statistically significant differences are shown in Supplementary Table 6. **(C)** Western Blot showing TRPV4 expression (band at ∼95 kDa) in WT and TRPV4-overexpressing cells revealed by anti-TRPV4 antibody and GFAP (band at ∼50 kDa) as astroglial marker. On the right, bar graph summarizing the densitometric analysis of TRPV4 relative quantification in *Aqp4*^–/–^ astrocytes knockdown for TRPV4 with siRNA and a scramble siRNA used as control. Data are expressed as means ± SEM and representative of three independent experiments (unpaired *t*-test, ****p < 0.0001). (For *Aqp4*^–/–^ with scramble: Ctrl with Ca^2+^ *n* = 10; 4αPDD with Ca^2+^ *n* = 9. For *Aqp4*^–/–^ with TRPV4-siRNA: Ctrl with Ca^2+^ *n* = 9; 4αPDD with Ca^2+^ *n* = 11; TG w/o Ca^2+^ *n* = 10).

Although 4αPDD can be regarded as a TRPV4 specific activator (Watanabe et al., 2002; Vriens et al., 2007; Butenko et al., 2012; Gao et al., 2019), we performed water transport measurements in primary cultured *Aqp4*^–/–^ astrocytes silenced for TRPV4 by a specific short-interference (si) siRNA (Figure 3). Scramble siRNA was used as control. Swelling assays in TRPV4 siRNA transfected astrocytes were conducted in the presence of 4αPDD in extracellular solution added with calcium and upon TG incubation in calcium-free extracellular medium. TRPV4 silencing was assessed through densitometric analysis on Western Blot data (Figure 3C) of lysates. GFAP detection was used as positive control for astrocyte culture purity.

**TABLE 2:** Time constants (τ) of the swelling phase of *Aqp4*^–/–^ astrocytes treated with TRPV4 and scramble siRNAs.

τ (swelling)	*Aqp4*^–/–^ + scramble siRNA	*Aqp4*^–/–^ + TRPV4 siRNA
Ctrl with extracellular calcium	8.956 s ± 0.3184	8.925 s ± 0.3440
+4αPDD with extracellular calcium	5.347 s ± 0.1784	8.480 s ± 0.4079
Thapsigargin + EGTA	–	5.930 s ± 0.2590

Data are shown as mean ± SEM.

As revealed by the analysis of the swelling kinetics (Figure 3B), the absence of TRPV4 induced by siRNA led to the impossibility, compared to controls, of augmenting water transport rates despite the stimulation with 4αPDD and the presence of external calcium. On the other hand, faster swelling rates were restored in cells knockdown for TRPV4 when intracellular calcium rise was promoted under TG stimulus. These results, therefore, (1) confirmed data obtained in native *Aqp4*^–/–^ astrocytes shown in Figure 2 by strengthening the functional role of TRPV4 in augmenting water transport (2) and provided evidence that calcium is involved in the founded AQP-independent modulation of cell swelling.

### 3.2. TRPV4 overexpression and activation promote faster volume changes in HEK-293 cells

**FIGURE 4:**
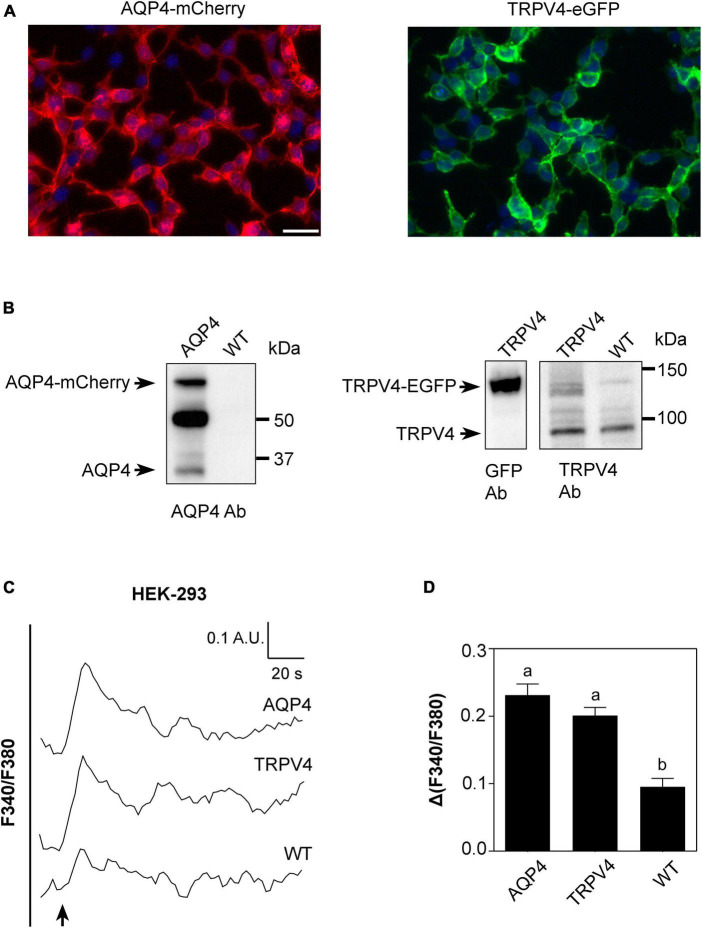
Characterization of AQP4-mCherry and TRPV4-eGFP stably transfected HEK-293 cells. **(A)** Epifluorescence images of AQP4-mCherry (red) and TRPV4-eGFP (green) expression in stably transfected HEK-293 cells. DAPI is in blue (scale bar 50 μm). **(B)** Western Blot analysis of AQP4 and TRPV4-overexpressing cells and WT, as indicated, revealed by anti-AQP4, anti-GFP, and anti-TRPV4 antibodies (Ab), respectively. Left: AQP4 expression in AQP4-overexpressing cells revealed as two bands at ∼60 kDa for recombinant AQP4 and at ∼32 kDa for untagged. No AQP4 expression was detected in WT cells. Right: TRPV4 expression revealed as one band at ∼130 kDa for recombinant TRPV4 in TRPV4-overexpressing cells with anti-GFP and anti-TRPV4 antibodies and one band at ∼95 kDa for native TRPV4 in both transfected and WT cells detected with anti-TRPV4 antibody. **(C)** Superimposed kinetics of calcium responses expressed as a ratio of fluorescence at 340 and 380 nm (F340/F380) under hypotonic stimulus performed by FlexStation3 in Fura2-AM loaded cells at 37°C. **(D)** Bar graph showing calcium amplitude [Δ(F340/F380)] for the three cell lines. Different letters indicate significant differences between the indicated cell lines. Data are reported as mean ± SEM and representative of three independent experiments (AQP4 = 0.2315 ± 0.01639, *n* = 25; TRPV4 = 0.2012 ± 0.01187, *n* = 22; WT = 0.09547 ± 0.01245, *n* =8). Brown-Forsythe ANOVA test and Tamhane’s T2 multiple comparisons test (*p* < 0.0001). Detailed statistically significant differences are shown in Supplementary Table 7.

Based on results shown in Figures 2, 3, we conducted a more detailed biophysical investigation of the augmented cellular swelling upon TRPV4 activation using HEK-293 cell line, shown to be a suitable cell model for water transport studies in fluorescent-based assays (Gao et al., 2005; Heo et al., 2008). With the aim to ascertain if the observed phenomenon is conserved in mammalian cells or exclusive to brain and determine the biophysical features of TRPV4-modulated water transport, HEK-293 cells were stably transfected with a GFP-tagged human TRPV4 cDNA sequence and with mCherry-tagged human AQP4 cDNA. Non-transfected cells were used as negative controls. Widefield fluorescence microscopy revealed the level of purity and protein expression of GFP or mCherry stable clones (Figure 4A). Western Blot analysis showed that stably transfected cells featured the predicted molecular weight for TRPV4-eGFP and AQP4-mCherry bands (∼130 and ∼60 kDa, respectively) and that HEK-293 cells do not natively express AQP4 but TRPV4 (band at ∼95 kDa) (Figure 4B). The anti-TRPV4 antibody used for the Western Blot recognizes the TRPV4 epitope at the C-term of the TRPV4 sequence. Being the EGFP tag located at the TRPV4-C-term, we hypothesized that the EGFP could affect the immunogenicity of the epitope recognized by the antibody. Western Blot revealed that the band for transfected TRPV4 was not as strong as expected. Therefore, TRPV4 HEK-293 clones were screened in Western Blot also by anti-GFP antibody (Figure 4B), which provided a positive and stronger signal for the recombinant protein, and eventually isolated by FACS cytometry (Supplementary Figure 3). Notably, a band at ∼32 kDa was also observed in AQP4-overexpressing cells, likely due to the expression of AQP4 without a fluorescent tag cleaved during sample denaturation, as previously reported (Rana et al., 2018).

Based on the evidence that functional AQP4 expression strongly increases TRPV4-mediated intracellular calcium signal following cell swelling (Benfenati et al., 2011; Mola et al., 2016), intracellular calcium response to a 60 mOsm/L gradient in Fura2-AM-loaded cells was used to assess the functional expression of both TRPV4 and AQP4 in the stably transfected cell lines (Figure 4C). Experiments were performed at 37°C, the temperature at which TRPV4 is thermally activated. Results showed that not only TRPV4 but also AQP4-overexpressing cells feature higher intracellular calcium increases than non-transfected WT cells, in line with previous data obtained in WT and *Aqp4*^–/–^ astrocytes (Mola et al., 2016; Figure 4D).

Altogether, these results show that (a) the two cell lines overexpress functional AQP4 and TRPV4 channels and that the tags do not affect protein functioning; (b) low levels of TRPV4 are also expressed in non-transfected WT cells; and (c) the contributions of TRPV4 and AQP4 to hypotonic shock-induced intracellular calcium signaling are comparable.

**FIGURE 5:**
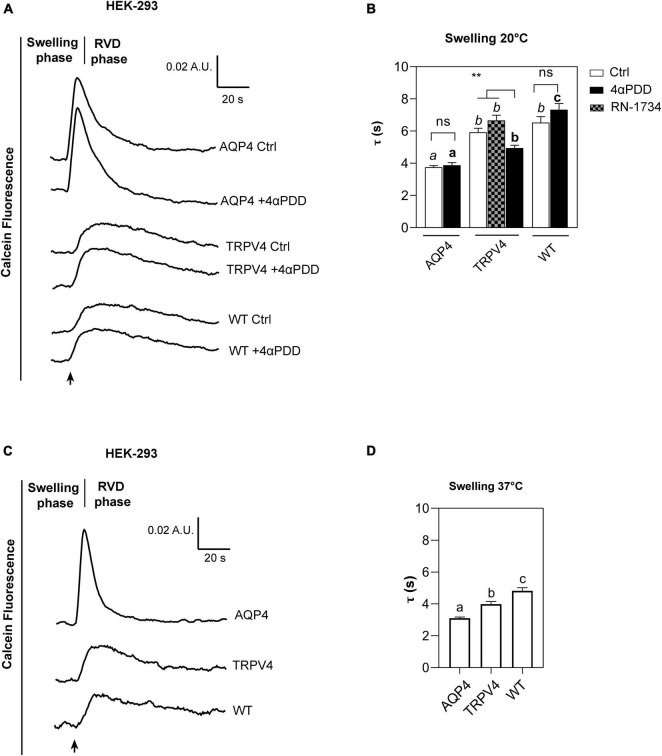
Calcein-AM quenching water transport assay in WT, AQP4, or TRPV4-overexpressing HEK-293 cells. **(A)** Representative water transport kinetics, showing swelling and RVD phase at 20°C under control conditions or stimulation by 10 μM 4αPDD. **(B)** Bar graph showing the time constant (τ) for the swelling phase at 20°C. Data are reported as mean ± SEM and representative of four independent experiments. For comparisons within each cell line: unpaired *t*-test with Welch’s correction (*ns*, *p* > 0.05; **p < 0.01) and Brown-Forsythe ANOVA test and Tamhane’s T2 multiple comparisons test between cell lines for TRPV4 at 20°C (*p* < 0.001) for comparisons per condition between cell lines: Brown-Forsythe ANOVA test and Tamhane’s T2 multiple comparisons test for controls at 20°C and (in *italics*, *ns*, *p* > 0.05; *p* < 0.0001) and activated cells (in *bold*, *p* < 0.0001). Detailed statistically significant differences are shown in Supplementary Tables 8–10 (WT: Ctrl *n* = 18; 4αPDD *n* = 19. TRPV4: Ctrl *n* = 24; 4αPDD *n* = 24; RN-1734 *n* = 9. AQP4: Ctrl *n* = 13; 4αPDD *n* = 9). **(C)** Representative water transport kinetics, showing swelling and RVD phase at 37°C. **(D)** Bar graph showing the time constant (τ) for the swelling phase at 37°C. Data are reported as mean ± SEM and representative of three independent experiments (WT: *n* = 18; TRPV4: *n* = 29; AQP4: *n* = 33). Brown-Forsythe ANOVA test and Tamhane’s T2 multiple comparisons test (*p* < 0.0001). Detailed statistically significant differences are shown in Supplementary Table 11.

The kinetics of cell volume changes under hypotonic conditions were measured with Calcein-AM quenching assay to assess the effect of TRPV4 overexpression on plasma membrane water fluxes (Figure 5). While AQP4 water channel is constitutively opened (Verkman, 2012), TRPV4 is activated by either warm temperatures ranging from 27°C to over 34°C (Chung et al., 2003; Shibasaki, 2020) or chemical agonist 4αPDD at lower temperatures (Butenko et al., 2012). Therefore, water transport assays were carried out: at 20°C in the presence or absence of 4αPDD as well as in presence of TRPV4 specific inhibitor RN-1734 (shown to completely inhibit both ligand- and hypotonicity-activated TRPV4) (Vincent et al., 2009), and at 37°C. Also, measurements at 20°C were aimed at minimizing the temperature-dependent lipid-mediated water permeation, while those performed at 37°C reproduced physiological conditions.

The non-linear regression fitting of water transport kinetics showed that AQP4 overexpressing cells performed the fastest swelling kinetics at 20 and 37°C (Figure 5 and Table 3), as expected. Interestingly, upon chemical and thermal activation of TRPV4, TRPV4-overexpressing cells performed faster swelling rates than RN-1734 inactivated TRPV4-overexpressing cells or controls. Although TRPV4 is endogenously expressed in HEK-293 (Figure 4B), it was not surprising that the exposure of WT cells to 4αPDD was not associated with increased water transport rates since native TRPV4 was found to be non-functional (Supplementary Figure 1).

**TABLE 3:** Time constants (τ) of the swelling phase of AQP4, TRPV4, and WT cells depending on TRPV4 activating conditions (*inactive TRPV4; #active TRPV4).

τ (swelling)	AQP4	TRPV4	WT
20°C (*)	3.757 s ± 0.09539	6.029 s ± 0.2393	6.391 s ± 0.4172
20°C + 4αPDD (#)	3.880 s ± 0.1660	5.156 s ± 0.2501	6.915 s ± 0.3845
37°C (#)	3.100 s ± 0.07283	3.983 s ± 0.1693	4.828 s ± 0.1872
20°C + 4αPDD + RN-1734 (*)	–	6.661 s ± 0.3300	–

Data are shown as mean ± SEM.

Faster swelling kinetics were overall observed at 37°C in each cell line compared to 20°C, due to the effects conferred by temperature, like the fluidity of the lipid bilayer or the reorganization and composition of membrane domains (Nickels et al., 2019; Ahmadi et al., 2022).

Further proving that water channels represent the fastest way for osmotic water movement across the plasma membrane, these results provide evidence of a positive effect of TRPV4 on plasma membrane water permeability of HEK-293 cell lines, as well as in brain astrocytes.

### 3.3. TRPV4 activation alters plasma membrane water transport biophysical parameters

**FIGURE 6:**
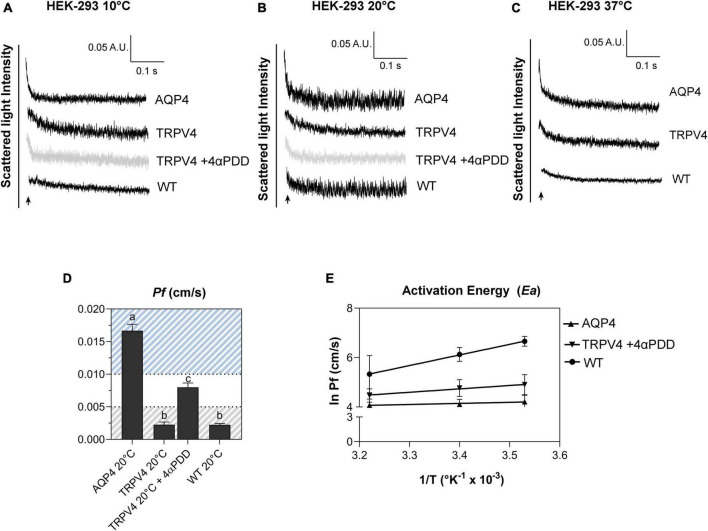
Biophysical parameters (*Pf* and *Ea*) associated with plasma membrane water transport in TRPV4-overexpressing cells measured by stopped-flow light scattering. **(A–C)** Representative curves for the time course of scattered light intensity at 10, 20, and 37°C in response to a 100 mOsm/L outwardly directed osmotic gradient resulting in cell swelling (decreased scattered light intensity). Data show AQP4 and TRPV4-expressing cells, where the TRPV4 channel was activated by 4αPDD agonist **(A,B)** or thermally **(C)**. Each of the time courses is plotted within the same y-range. **(D)** Osmotic water permeability coefficient (*Pf*) computed from kinetics at 20°C. The ranges of *Pf* values that predict facilitated water movement by molecular pores (>0.01 cm/s) or consistent with water diffusion across a lipid portion of a membrane (<0.005 cm/s) are evidenced in the bar plot with blue and gray lines, respectively. Data are represented as the mean ± SEM and representative of three independent experiments. Brown-Forsythe ANOVA test and Tamhane’s T2 multiple comparisons test (*p* < 0.0001). Detailed statistically significant differences are shown in Supplementary Table 12. **(E)** Arrhenius plot of temperature-dependence data for the three cell lines, as indicated.

The biophysical properties of the water movement across the plasma membrane of TRPV4-overexpressing cells were defined using the osmotic permeability coefficient (*Pf*) and the Arrhenius activation energy (*Ea*, kcal/mol) computed from the rate constants obtained with stopped-flow light scattering assay (Mola et al., 2009). Both parameters allows distinguishing between water flows occurring via simple diffusion *versus* facilitated transport (Figure 6).

Results obtained from AQP4, TRPV4-overexpressing cells, and WT confirmed that TRPV4 activation, induced by both agonist and temperature, contributes to the increase in cell swelling rate in the absence of AQP4 (Figures 6A–C). The osmotic water permeability coefficient (*Pf*) (cm/s) (Table 4) computed from the analysis of the rate constants indicated that TRPV4 opening significantly enhances water membrane permeability in HEK-293 cells overexpressing TRPV4, albeit to a lesser extent than the expression of AQP4 (Figure 6D).

**TABLE 4:** Osmotic permeability coefficients (*Pf*) for AQP4, TRPV4, and WT cells at 20°C.

*Pf* (cm/s)	AQP4	TRPV4	WT
20°C	0.01667 ± 0.0007	0.002261 ± 0.0004	0.002249 ± 0.0002
20°C + 4αPDD	–	0.007985 ± 0.0006	–

Temperature dependence experiments were conducted to determine the activation energy (*Ea*) associated with plasma membrane water transport in TRPV4 overexpressing cells. Figure 6E summarizes the calculated *Pf* values at three different temperatures in the form of an Arrhenius plot, where the slope multiplied by the gas constant R (kcal/mol) is proportional to the activation energy (Verkman, 2000; Solenov et al., 2004). The activation energy value deduced for WT (8.532 ± 0.4 kcal/mol) and AQP4-expressing cells (0.860 ± 0.4 kcal/mol) confirmed a strong and weak temperature-dependence for water movement and were consistent with a lipid diffusion and aqueous channel-mediated model, respectively (Verkman, 2000). Of note, the *Ea* of AQP4 cells was found to be even lower than the reference ranges, likely due to the significant overexpression of the water channel in the cell line. Interestingly, the *Ea* computed for agonist activated-TRPV4 cells (2.728 ± 1.0 kcal/mol) was indicative of a facilitated pathway for water movement across the plasma membrane (Verkman, 2000).

**FIGURE 7:**
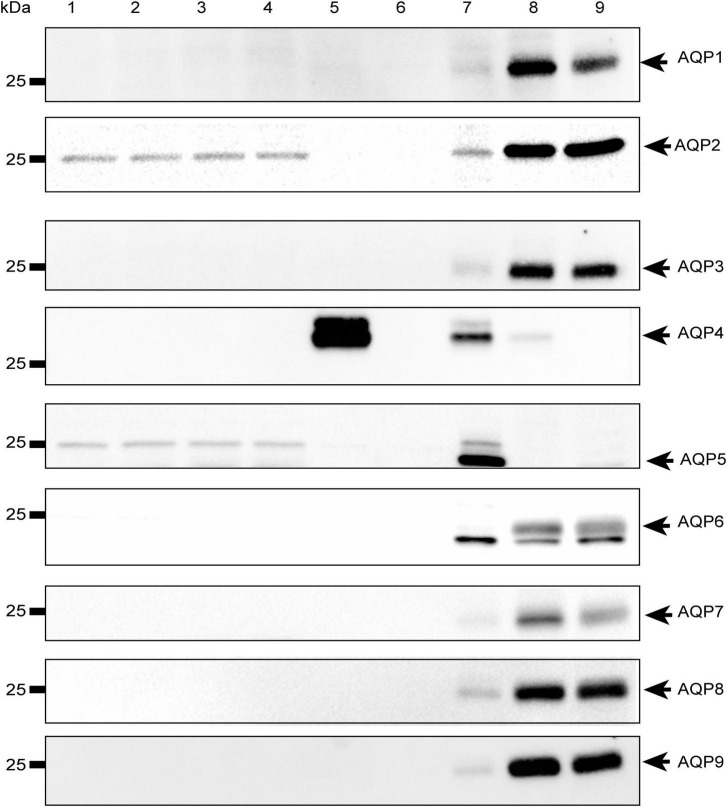
Western-Blot analysis of aquaporins in mouse astrocytes and HEK-293 cells. Western blot analysis of aquaporins (from 1 to 9) in WT and TRPV4-overexpressing HEK-293 cells and WT and *Aqp4*^–/–^ astrocytes. Protein samples (10 μg) were loaded as follows: lane 1-2, HEK-293 WT; lane 3-4, TRPV4-overexpressing HEK-293; lane 5, WT astrocytes; lane 6 *Aqp4*^–/–^ astrocytes. For WB of AQP1, AQP3, AQP4, AQP6-AQP9: lane 7, mouse brain; lane 8, mouse kidney; lane 9, mouse liver. For WB of AQP2: lane 7, mouse kidney; lane 8 (2.5 μg) and lane 9 (5 μg), M1 mouse kidney cortical collecting duct cells stably transfected with human AQP2 (MCD4) for AQP2 (Milano et al., 2018). For WB of AQP5: lane 7, mouse lung; lane 8, mouse kidney; lane 9, mouse liver. Coomassie Blue staining was used as loading control for Western Blot (Supplementary Figure 4).

To rule out the possibility that the observed effect was due to other water channels, we investigated the expression of the most abundant aquaporins in mammalian systems (AQP1-9) in both HEK-293 cells and mouse brain astrocytes by Western Blot. We found that the aforementioned aquaporins are expressed neither in HEK-293 cells nor in brain astrocytes or, if so, they are present below detectable levels under our experimental conditions, except for AQP4 in WT astrocytes and, interestingly, AQP2 in both WT and TRPV4-overexpressing HEK-293 cells (Figure 7). Of note, the faint band visible at ∼25 kDa in the blot is not specific for AQP5, whose specific band is indicated by the arrow at ∼22 kDa and identified in the lung, but in no other sample, including HEK-293 cells as previously reported (Sidhaye et al., 2006; Alam et al., 2016).

## 4. Discussion

Reduced AQPs expression has been reported under pathological conditions (Markou et al., 2022), including brain edema after stroke or trauma (Papadopoulos et al., 2002; Clément et al., 2020; Dadgostar et al., 2022), neurological diseases (Yang et al., 2011; Alvestad et al., 2013; Li et al., 2022), cancer progression and metastasis (Lan et al., 2022), and inflammation. Of note, AQP1 and AQP4 are reported to be downregulated in the choroid plexus and the ependyma, respectively, of spontaneously hypertensive rat, as a putative compensatory mechanism to reduce the cerebrospinal fluid (CSF) production and volume (Carnero Contentti and Correale, 2021; González-Marrero et al., 2022). A reduction in AQP4 levels and mislocalization is also observed in aging mice or upon traumatic brain injury, thus explaining the impairment of glymphatic system and solutes clearance reported under these conditions (González-Marrero et al., 2022; Salman et al., 2022).

The major findings in this study relate to the unconventional role of the ion channel TRPV4 in augmenting water transport in the absence of AQP4.

Aquaporins (AQPs) represent the primary path for water flows across cell membranes. However, it has been proposed that unconventional AQP-independent ways for water permeation exist and are putatively represented by cotransporters, membrane proteins that cotransport water together with solutes (MacAulay, 2021). There is evidence that TRPV4 is directly involved in water permeation since studies in retinal Müller cells showed that its activation is associated with faster cellular swelling in the absence of AQP4 (Jo et al., 2015) and TRPV4 inhibition prevents increased water diffusion in the retina of diabetic mice (Ríos et al., 2019). Moreover, TRPV4 may play a role in determining the resting membrane potential (Vm) and contributes to the modulation of Vm changes during the regulatory volume decrease (RVD) response (Netti et al., 2017).

This study shows that the fastest water transport rates for swelling are achieved in the presence of the AQP4 water channel. However, TRPV4 activation facilitates cellular swelling approximately twofold in the absence of AQP4. Compared to previous works, we here show that this phenomenon is conserved since similar effects previously reported in Muller cells (Jo et al., 2015) are detectable also in mouse astrocytes and embryonic kidney-derived HEK-293 cell line. Moreover, we shed light on a possible mechanism likely involving calcium-tunable and non-aquaporin-like pathway. The specificity of the TRPV4 effect on plasma membrane water transport was also proved by TRPV4 knockdown experiments in *Aqp4*^–/–^ astrocytes and TRPV4 chemical inhibition in HEK-293 cells. These results provided further and strong evidence that in the absence of both AQP4 and TRPV4 (TRPV4 siRNA in *Aqp4*^–/–^ astrocytes) cell swelling rates are reduced unless calcium rise is promoted from intracellular store depletion. Thapsigargin-induced calcium store depletion is also known to activate store-operated Ca^2+^ entry (SOCE) mechanism in the presence of external calcium through the SOCE activators, STIM1 and STIM2, and the Orai channels (Motiani et al., 2010; Frisch et al., 2019). However, given that no significant differences in water transport rates were found when different calcium sources were activated, calcium dynamics were not investigated further. Instead, the biophysical properties of water transport conferred by TRPV4 were quantified.

We hypothesize that the calcium entering either through the opened TRPV4, or following ER depletion and ER replenishment activates secondary pathways which, in turn, mediate augmented water transport. This putative mechanism arises from the evidence that (1) only when TRPV4 is opened and calcium intake is promoted from external medium, faster water transport kinetics are recorded; (2) no similar effects were obtained upon TRPV4 activation in calcium-free external solution (Figure 2B); and (3) augmented water transport kinetics were registered upon calcium release from intracellular stores.

The hypothesis of a TRPV4-tunable permeation pathway for water transport occurring through the putative wet ion channel VRAC was also investigated based on studies showing that plasma membrane water permeability is decreased by VRAC inhibition (Nilius, 2004; Supplementary Figure 2). Non-linear regression of water transport kinetics (Supplementary Table 1) showed that VRAC knockdown does not alter the swelling rate in both TRPV4-overexpressing and WT cells, while the effect of TRPV4 on increasing the plasma membrane water permeability occurs despite of VRAC knockdown (Supplementary Figures 2D, E). Therefore, despite not excluding that VRAC could provide additional permeation pores for water as an ion channel, we show that this is not dependent or tunable by TRPV4 in the context here analyzed.

Interestingly, we found that TRPV4 modulation changes the plasma membrane water transport biophysical parameters (*Pf* and *Ea*). Considering *Pf* > 0.01 cm/s for AQP-mediated and *Pf* < 0.005 cm/s for lipid-mediated water transport at 25–37°C (Verkman, 2000) as reference values, WT (*Pf* = 0.002249 ± 0.0002, at 20°C) and non-activated TRPV4 cells (*Pf* = 0.002261 ± 0.0004, at 20°C) exhibit simple diffusion features and AQP4 cells (*Pf* = 0.01667 ± 0.0007, at 20°C) water channel-mediated characteristics, as expected. Interestingly, *Pf* computed under TRPV4 activating conditions (*Pf* = 0.008 ± 0.0006) was found to be in a non-fully defined interval. In fact, *Pf* measured in TRPV4 cells exposed to 4αPDD is neither compatible with aquaporin-mediated water flows nor consistent with water diffusion through membranes. Of note, absolute values of *Pf* are not determinant *per se*, but rather the ranges within it falls (i.e., below 0.005 cm/s or above 0.01 cm/s). On the other hand, the low activation energy computed for agonist-activated TRPV4 cells (2.73 ± 1.9 kcal/mol) was more indicative of a channel-mediated pathway for water movement across the plasma membrane (considering *Ea* 3–5 kcal/mol for water movement through channels) (Verkman, 2000).

Western blot analysis of aquaporins revealed that WT astrocytes selectively express AQP4 even though other AQPs different from AQP4 have been reported (Gao et al., 2012; Hirt et al., 2018; Zhou et al., 2022). Moreover, no other AQPs were upregulated in *Aqp4*^–/–^ astrocytes. Different AQPs (AQP1, AQP3, AQP4, AQP5, AQP8, and AQP9) were shown to be expressed in astrocytes (Zhou et al., 2022). Among them, AQP1 has been mostly associated with neuropathological conditions (Potokar et al., 2016) and found in human but not mouse and rat astrocytes (Gao et al., 2012), while AQP9 has been identified in primary cultured mouse astrocytes (Wang et al., 2015), brain tissue (Badaut et al., 2001, 2004; Mylonakou et al., 2009) and, more extensively, in primary astrocytes prepared from neurospheres (Cavazzin et al., 2006; Badaut et al., 2012, 2004) under physiological conditions. However, contrasting evidence had ruled out the expression of AQP9 in brain or astrocytes (Elkjar et al., 2000; Rojek et al., 2007) and some works have suggested that AQP9 signal is sometimes undetectable due to the very low levels of AQP9 mRNA in the brain (Mylonakou et al., 2009) that is only 3% (in rat) and even less than 1% (in mouse) of the concentration of AQP9 mRNA in liver, thus offering a plausible explanation of why AQP9 could not been revealed in our Western Blot data.

AQP2 was instead found to be expressed in HEK-293, both in WT and TRPV4-overexpressing cells. AQP2 is physiologically found in the collecting duct of the kidney and expressed in the apical membrane under ADH-hormone stimulus (Wilson et al., 2013). Vasopressin binds to V2 receptors on the basolateral membrane of principal cells and stimulates the fusion of vesicles enriched in AQP2 with the plasma membrane in a cAMP-dependent way (Valenti et al., 2005). If, in principle, AQP2 might enhance water transport rates in HEK-293 cells *per se*, whether this occurs upon TRPV4 and calcium modulation would require further investigation. Given the absence of AQP2 in brain astrocytes (Figure 7), it is likely that other calcium-dependent mechanisms common with astrocytes and HEK-293 cells are tuned. However, they were not investigated further since the main interest of this study was to dissect the specific role of AQP4, here addressed with *Aqp4*^–/–^ astrocytes. However, if mediated by any AQPs, we would expect that the *Pf* fell within the interval of water channel-mediated water transport which we did not find, in line with molecular data.

Based on such evidence, although alternative mechanisms acting on the permeability of the lipid bilayers cannot be excluded, we speculate that TRPV4 tunes the functionality of calcium-regulated aqueous channels with large pores to transport water (Graphical abstract), such as Na-K-Cl cotransporter-1 (NKKC-1). Proposed to be a cotransporter of water (Hamann et al., 2010), NKCC-1 was found to be associated with increased cellular swelling when activated (Kintner et al., 2002; Kahle et al., 2009; Jayakumar et al., 2011) and with significantly impaired cell swelling when inhibited or absent (Su et al., 2002). Interestingly, it has also been reported that NKCC-1 can be activated by TRPV4 via calcium accumulation (Toft-Bertelsen et al., 2022). In support of this, NKCC-1 was found to be expressed in astrocytes (Zhang et al., 2017) and HEK-293 cells (Haas and Sontheimer, 2010). In this view, future studies could aim at testing the existence of a TRPV4/NKCC-1 interplay or look for other potential interactors of TRPV4, including other members of the Na-K-Cl cotransporters (NKCC). If the hypothesis of TRPV4/NKCC-1 interplays was demonstrated, we would anticipate that the water transport is not bidirectional. Instead, we would expect that, as formulated by MacAulay (2021) the cotransport of water along with Na^+^, K^+^, and Cl^–^ intake in the cell would be predominant and independent of the transmembrane osmotic gradient.

Therefore, we here show that although the modulation of water transport dynamics is massively dependent on the expression of water channels, AQP-alternative pathways for water permeation exist and are likely to be tuned in a TRPV4/calcium-dependent manner independently of its source or way of entry to the cell.

Intracellular calcium events are physiologically generated in cells or cellular domains that lack AQPs but express TRPV4 and here regulate different key cellular functions such as in PAPs (peri-synaptic processes) of brain astrocytes where AQP4 is expressed at very low concentrations (Butenko et al., 2012; Nagelhus and Ottersen, 2013), or even in other CNS cells, like neurons, which do not express aquaporins but TRPV4 (Zhang and Verkman, 2010). In the perisynaptic astrocytic processes (PAPs) (Lavialle et al., 2011), calcium modulates pathways for astrocyte signaling and functionality (i.e., gliotransmitter release and uptake; synaptic plasticity; modulation of brain circuits; ion and water homeostasis) (Guerra-Gomes et al., 2018; Aboufares El Alaoui et al., 2021; Lia et al., 2021).

As shown in the present study, the mild effect of TRPV4 on plasma membrane water permeability is overall negligible in the presence of AQP4, whose effect is major. This data has been obtained from primary astrocytes in *in vitro* cultures where AQP4 and TRPV4 are co-expressed. Physiologically, this situation can resemble that typical of astrocyte perivascular microdomains where TRPV4 is co-expressed with abundant levels of AQP4 channels which, therefore, likely hide TRPV4 contribution to the water transport rate. However, if this correctly indicates that TRPV4 does not play a primary role when AQP4 and TRPV4 are equally abundant and co-expressed, this does not rule out the possibility for TRPV4 to play a role under physiological conditions when AQP4 is absent or TRPV4/AQP4 ratio is high. On the other hand, considering that AQP9 is expressed in astroglia (Badaut et al., 2001, 2004, 2012; Cavazzin et al., 2006; Mylonakou et al., 2009; Wang et al., 2015) and AQP1 can be upregulated in response to stress factors or in pathological states (Potokar et al., 2016), our experimental conditions do not allow to rule out the contribution of other AQPs to the water transport rate of astrocyte plasma membrane *in vivo*.

One possible understanding of the patho-physiological significance of the observed rapid (i.e., lasting minutes) TRPV4/calcium-regulated cellular swelling is that where low amounts or no aquaporins are expressed, a finely tunable increase in water permeability might be required alternatively to the fast aquaporin-mediated water transport. We hypothesize that this might be used as a protective tool for ensuring the integrity of cells and membranes or as a back-up mechanism to sustain solutes clearance or limit the fluid movement within the brain thus maintaining ion and water homeostasis in the brain.

## 5. Conclusion

In conclusion, this study demonstrates for the first time that the calcium rise deriving from TRPV4 or intracellular calcium stores can influence plasma membrane water permeability. These data, therefore, support the recent hypothesis that alternative ways for water permeation exist, such as the co-transport of water occurring through membrane transporters (MacAulay, 2021) that will need to be furtherly investigated and identified in future studies. The discovery of a new mechanism that enhances water transport in the absence of water channels contributes to a deeper understanding of cell swelling and lays the ground for the targeting of alternative pathways tuned in pathological conditions, where uncontrolled brain swelling occurs (Rungta et al., 2015).

## Data availability statement

The original contributions presented in the study are included in the article/Supplementary material, further inquiries can be directed to the corresponding author.

## Author contributions

BB, MM, FF, MC, VB, and GN: conceptualization, validation, and investigation. BB, MM, FF, ES, CG, AC, GM, AF, and GN: methodology and formal analysis. BB, MM, FF, ES, and CG: data curation. BB and GN: writing—original draft preparation. BB, MM, FF, AF, MC, VB, and GN: writing—review and editing. GN: supervision and project administration. GN, AF, and VB: funding acquisition. All authors have read and agreed to the published version of the manuscript.

## Footnotes

1
www.euroclonegroup.it

2
www.lgcstandards-atcc.org

3
www.thermoscientific.com

4
http://www.merckmillipore.com/

5
http://www.bio-rad.com

6
http://www.moleculardevices.com
