# Supplementary material for: AQP4-independent TRPV4 modulation of plasma membrane water permeability
Source: Front Cell Neurosci. 2023 Aug 31;17:1247761. doi: 10.3389/fncel.2023.1247761 (PMC10500071; doi:10.3389/fncel.2023.1247761)
Supplement: Supplementary file 1 [file Table_1.pdf]

Supplementary Material

Supplementary Tables

Supplementary Table 1. Time constants (τ) of the swelling phase of scramble and LRRC8/A siRNA-treated TRPV4 and WT cells under different TRPV4 activating conditions (*inactive TRPV4; #active TRPV4) (Supplementary Figure 2). Data are shown as mean ± SEM.

**τ (swelling)**	**TRPV4 + scramble siRNA**	**TRPV4 + LRRC8/A siRNA**	**WT + scramble siRNA**	**WT + LRRC8/A siRNA**
20°C (*)	6.371 s ± 0.2345	6.140 s ± 0.2461	6.138 s ± 0.3446	6.605 s ± 0.4370
20°C +4αPDD (#)	4.631 s ± 0.2205	4.567 s ± 0.3696	6.435 s ± 0.3472	5.918 s ± 0.4145

Supplementary Table 2. Percentage of cell volume recovery (%) of scramble and LRRC8/A siRNA-treated TRPV4 and WT cells under different TRPV4 activating conditions (*inactive TRPV4; #active TRPV4) (Supplementary Figure 2). Data are shown as mean ± SEM.

**Recovery (%)**	**TRPV4 + scramble**	**TRPV4 +LRRC8/A siRNA**	**WT + scramble**	**WT + LRRC8/A siRNA**
20°C (*)	80.86 ± 2.885	46.10 ± 6.680	80.08% ± 7.039	43.92 ± 10.35
20°C +4αPDD (#)	78.59 ± 3.779	44.20 ± 5.683	79.32 ± 4.946	36.26 ± 10.30

Supplementary Table 3. Kruskal-Wallis with Dunn's multiple comparison test for aqp4-/- astrocytes for comparisons between conditions (Figure 2).

aqp4-/- TG w/o Ca^{2+} vs. aqp4-/- TG + 4αPDD	ns
aqp4-/- TG w/o Ca^{2+} vs. aqp4-/- 4αPDD w/o Ca^{2+}	**
aqp4-/- TG w/o Ca^{2+} vs. aqp4-/- 4αPDD with Ca^{2+}	ns
aqp4-/- TG w/o Ca^{2+} vs. aqp4-/- Ctrl w/o Ca^{2+}	*
aqp4-/- TG w/o Ca^{2+} vs. aqp4-/- Ctrl with Ca^{2+}	*
aqp4-/- TG + 4αPDD vs. aqp4-/- 4αPDD w/o Ca^{2+}	*
aqp4-/- TG + 4αPDD vs. aqp4-/- 4αPDD with Ca^{2+}	ns
aqp4-/- TG + 4αPDD vs. aqp4-/- Ctrl w/o Ca^{2+}	*
aqp4-/- TG + 4αPDD vs. aqp4-/- Ctrl with Ca^{2+}	*
aqp4-/- 4αPDD w/o Ca^{2+} vs. aqp4-/- 4αPDD with Ca^{2+}	***
aqp4-/- 4αPDD w/o Ca^{2+} vs. aqp4-/- Ctrl w/o Ca^{2+}	ns
aqp4-/- 4αPDD w/o Ca^{2+} vs. aqp4-/- Ctrl with Ca^{2+}	ns
aqp4-/- 4αPDD with Ca^{2+} vs. aqp4-/- Ctrl w/o Ca^{2+}	**
aqp4-/- 4αPDD with Ca^{2+} vs. aqp4-/- Ctrl with Ca^{2+}	**
aqp4-/- Ctrl w/o Ca^{2+} vs. aqp4-/- Ctrl with Ca^{2+}	ns

Supplementary Table 4. Kruskal-Wallis with Dunn's multiple comparison test for comparison between astrocyte genotypes under control conditions (Figure 2).

aqp4-/- 4αPDD w/o Ca^{2+} vs. WT Ctrl w/o Ca^{2+}	***
aqp4-/- 4αPDD w/o Ca^{2+} vs. WT 4αPDD w/o Ca^{2+}	***
aqp4-/- 4αPDD w/o Ca^{2+} vs. WT Ctrl with Ca^{2+}	***

aqp4-/- Ctrl w/o Ca^{2+} vs. WT Ctrl w/o Ca^{2+}	**
aqp4-/- Ctrl w/o Ca^{2+} vs. WT 4αPDD w/o Ca^{2+}	***
aqp4-/- Ctrl w/o Ca^{2+}+ vs. WT Ctrl with Ca^{2+}	**
aqp4-/- Ctrl with Ca^{2+}+ vs. WT Ctrl w/o Ca^{2+}	**
aqp4-/- Ctrl with Ca^{2+} vs. WT 4αPDD w/o Ca^{2+}	***
aqp4-/- Ctrl with Ca^{2+} vs. WT Ctrl with Ca^{2+}	**

Supplementary Table 5. One-way ANOVA with Newman-Keuls multiple comparisons test for comparisons between astrocyte genotypes under activating conditions (Figure 2).

aqp4-/- TG w/o Ca^{2+} vs. wt 4αPDD with Ca^{2+}	***
aqp4-/- TG w/o Ca^{2+}+ vs. wt TG + 4αPDD	**
aqp4-/- TG w/o Ca^{2+}+ vs. wt TG w/o Ca2+	***
aqp4-/- TG + 4αPDD vs. wt 4αPDD with Ca^{2+}	****
aqp4-/- TG + 4αPDD vs. wt TG + 4αPDD	****
aqp4-/- TG + 4αPDD vs. wt TG w/o Ca^{2+}	****
aqp4-/- 4αPDD with Ca^{2+} vs. wt 4αPDD with Ca^{2+}	**
aqp4-/- 4αPDD with Ca^{2+}+ vs. wt TG + 4αPDD	**
aqp4-/- 4αPDD with Ca^{2+} vs. wt TG w/o Ca^{2+}	**

Supplementary Table 6. Kruskal-Wallis with Dunn’s multiple comparison test for comparison between conditions in aqp4-/- astrocytes treated with TRPV4 siRNA and Scramble (Figure 3).

Scramble Ctrl with Ca^{2+} vs. Scramble +4αPDD with Ca^{2+}	****
Scramble Ctrl with Ca^{2+} vs. TRPV4 siRNA +4αPDD with Ca^{2+}	ns
Scramble Ctrl with Ca^{2+} vs. TRPV4 siRNA Ctrl with Ca^{2+}	ns
Scramble Ctrl with Ca^{2+} vs. TRPV4 siRNA TG w/o Ca^{2+}	***
Scramble +4αPDD with Ca^{2+} vs. TRPV4 siRNA +4αPDD with Ca^{2+}	**
Scramble +4αPDD with Ca^{2+} vs. TRPV4 siRNA Ctrl with Ca^{2+}	***
Scramble +4αPDD with Ca^{2+} vs. TRPV4 siRNA TG w/o Ca^{2+}	ns
TRPV4 siRNA +4αPDD with Ca^{2+} vs. TRPV4 siRNA Ctrl with Ca^{2+}	ns
TRPV4 siRNA +4αPDD with Ca^{2+}vs. TRPV4 siRNA TG w/o Ca^{2+}	*
TRPV4 siRNA Ctrl with Ca^{2+} vs. TRPV4 siRNA TG w/o Ca^{2+}	**

Supplementary Table 7. Brown-Forsythe ANOVA test and Tamhane's T2 multiple comparisons test for comparisons of calcium amplitude (Δ(F340/F380)) between HEK-293 cell lines (Figure 4).

WT VS. AQP4	****
WT VS. TRPV4	****
AQP4 VS. TRPV4	ns

Supplementary Table 8. Brown-Forsythe ANOVA test and Tamhane's T2 multiple comparisons test between swelling time constants of HEK-293 cells overexpressing TRPV4 at 20°C in the presence of 4αPDD or RN-1734 (Figure 5)

TRPV4 CTRL VS. TRPV4 + 4αPDD	**
TRPV4 CTRL VS. TRPV4 + RN-1734	ns
TRPV4 + 4αPDD VS. TRPV4 + RN-1734	**

Supplementary Table 9. Brown-Forsythe ANOVA test and Tamhane's T2 multiple comparisons test between swelling time constants of HEK-293 cell lines under control or inactivating TRPV4 conditions with RN-1734 at 20°C (Figure 5).

WT CTRL VS. TRPV4 CTRL	ns
WT CTRL VS. TRPV4 +RN-1734	ns
WT CTRL VS. AQP4 CTRL	****
TRPV4 CTRL VS. TRPV4 +RN-1734	ns
TRPV4 CTRL VS. AQP4 CTRL	****
TRPV4 +RN-1734 VS. AQP4 CTRL	**

Supplementary Table 10. Brown-Forsythe ANOVA test and Tamhane's T2 multiple comparisons test between swelling time constants in HEK-293 cell lines at 20°C + 4αPDD (Figure 5).

WT + 4αPDD VS. TRPV4 + 4αPDD	****
WT + 4αPDD VS. AQP4 + 4αPDD	****
TRPV4 + 4αPDD VS. AQP4 + 4αPDD	***

Supplementary Table 11. Brown-Forsythe ANOVA test and Tamhane's T2 multiple comparisons test between cell lines at 37°C (Figure 5).

WT VS. TRPV4	*
WT VS. AQP4	****
TRPV4 VS. AQP4	***

Supplementary Table 12. Brown-Forsythe ANOVA test and Tamhane's T2 multiple comparisons test for comparison between *Pf* values (Figure 6).

AQP4 20°C VS. TRPV4 20°C	****
AQP4 20°C VS. TRPV 20°C + 4αPDD	****
AQP4 20°C VS. WT 20°C	****
TRPV4 20°C VS. TRPV 20°C + 4αPDD	****
TRPV4 20°C VS. WT 20°C	ns
TRPV 20°C + 4αPDD VS. WT 20°C	***
