# Supplementary material for: AQP4-independent TRPV4 modulation of plasma membrane water permeability
Source: Front Cell Neurosci. 2023 Aug 31;17:1247761. doi: 10.3389/fncel.2023.1247761 (PMC10500071; doi:10.3389/fncel.2023.1247761)
Supplement: Supplementary file 2 [file Image_1.pdf]

Supplementary Material

1 Supplementary Figures

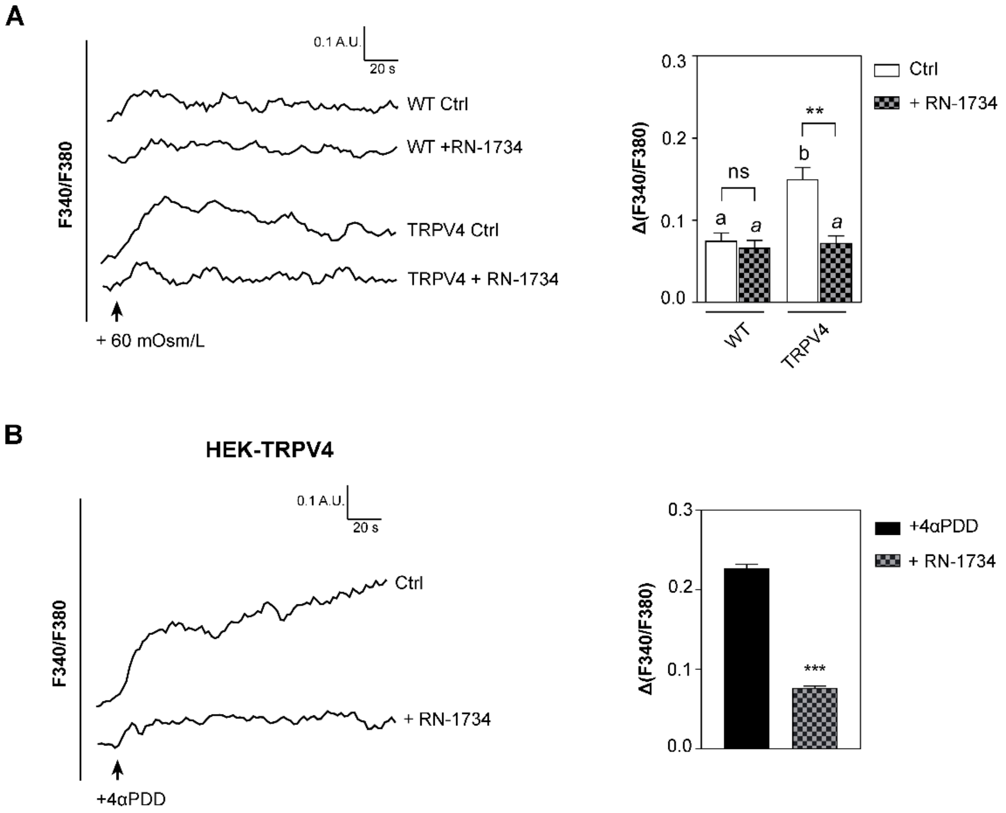

Supplementary Figure 1. (**A**) Intracellular calcium responses in WT and TRPV4-transfected HEK cells. (Left) Representative traces of hypotonicity-induced Ca2+ increase recorded in Fura2-loaded cells treated with 4αPDD (Ctrl) or with 4αPDD +TRPV4 antagonist RN1734. The arrow indicates the switch in the external osmolarity. (Right) Quantitative analysis of Ca2+ responses in the two cell lines. Data represent the mean values ± SEM of the Ca2+ amplitude (Δ(F340/F380)) obtained from three independent experiments (WT Ctrl = 0.07±0.0095, n=12; WT+RN-1734= 0.066±0.0088, n=16; TRPV4 Ctrl= 0.15±0.014, n=13; TRPV4+RN-1734= 0.07±0.009, n=17). Unpaired t-test for comparison between genotypes under the same conditions and between conditions of different genotypes (ns, $p>0.05$; ** $p<0.01$). (**B**) Intracellular calcium responses in TRPV4-transfected HEK cells. (Left) Representative traces recorded in Fura2-loaded cells exposed to 4αPDD (Ctrl) or to 4αPDD +TRPV4 antagonist RN1734 under isotonic conditions. The arrow indicates the addition of the TRPV4 agonist. (Right) Bar graph showing calcium amplitude (Δ(F340/F380)) obtained from three

independent experiments. Data represent the mean values ± SEM (Ctrl = 0.07±0.0033, n=11; WT+RN-1734= 0.22±0.0088, n=15. Unpaired t-test (p<0.001).

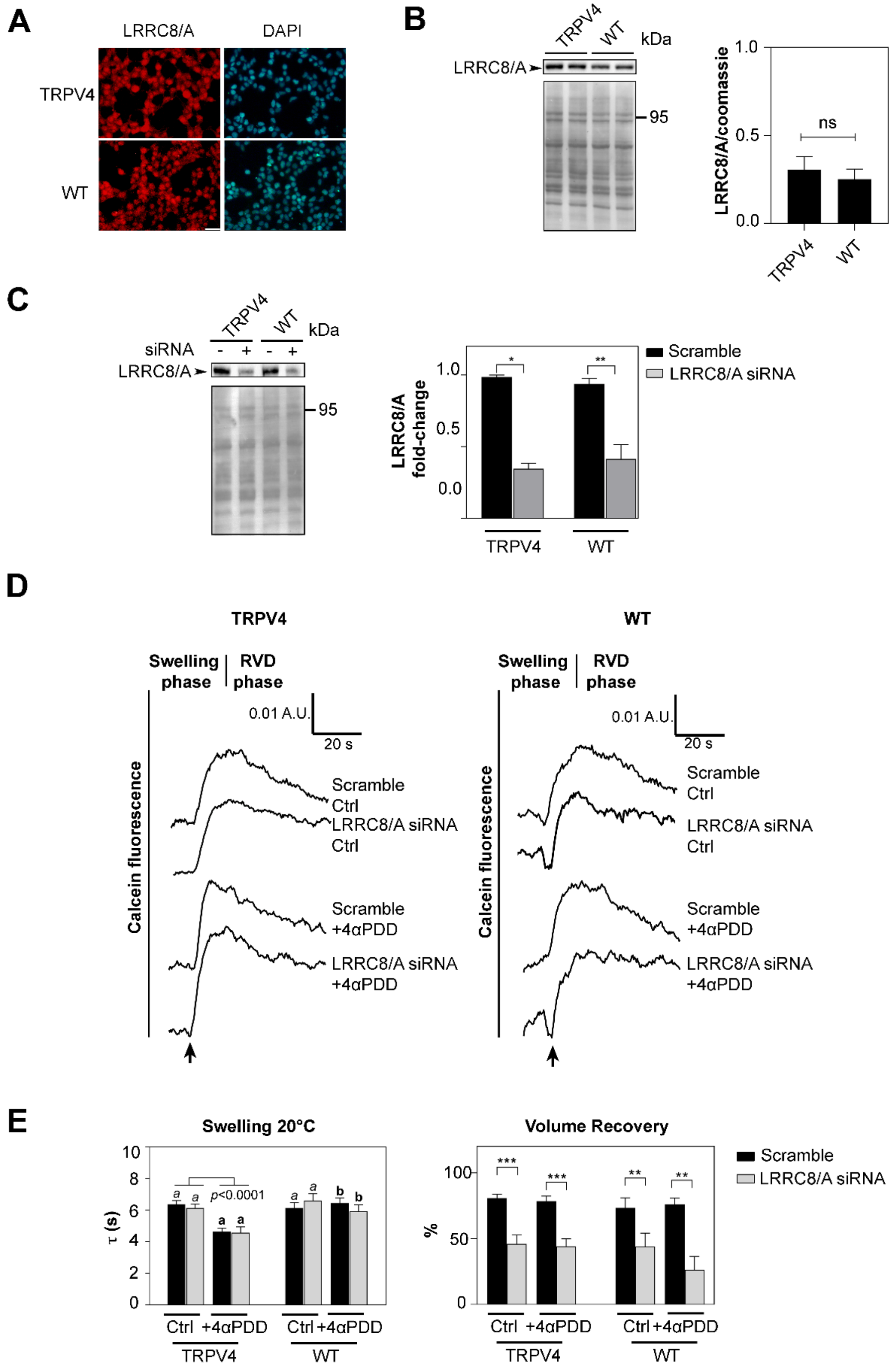

Supplementary Figure 2. (**A**) Immunofluorescence images of VRAC (red) expression in WT and TRPV4-overexpressing (TRPV4) cells. DAPI is in blue (scale bar 50 µm). (**B**) Western Blot showing

VRAC expression (band at ~95 kDa) in WT and TRPV4-overexpressing cells revealed by anti-LRRC8/A antibody. On the right, bar graph summarizing the densitometric analysis of VRAC relative quantification on Coomassie Blue staining of total proteins shown below. Data are expressed as means ± SEM (Mann-Whitney test; ns, $p>0.05$) and representative of five independent experiments. (**C**) Western blot analysis of VRAC expression in WT and TRPV4-overexpressing cells knockdown for VRAC with a specific LRRC8/A siRNA (+) and a scramble siRNA (-) used as control. The bar graph on the right shows the fold-change in the expression of VRAC-relative bands normalized on Coomassie Blue staining of total protein shown below. Data are expressed as means ± SEM and representative of three independent experiments (unpaired t-test, *$p<0.05$, **$p<0.01$). (**D**) Representative water transport kinetics recorded by Calcein-AM quenching assay upon 60 mOsm/L hyposmotic shock, showing swelling and RVD phase in TRPV4 overexpressing and WT cells treated with LRRC8/A siRNA (siRNA) and the control siRNA (scramble) at 20°C, in the presence (+4αPDD) or absence of the TRPV4 activator 4αPDD. (**E**) Bar graphs showing the time constant (τ) for the swelling phase (upper panel) and cell volume recovery ability expressed in percentage (%) (lower panel) recorded at 20°C in the presence (+4αPDD) or absence (Ctrl) of the TRPV4 activator 4αPDD. The silencing of LRRC8/A subunit reduced the ability of cell volume recovery after the hypotonic shock in cells compared to controls, as previously reported (Formaggio et al., 2019). Data are reported as mean ± SEM and representative of three independent experiments. For the swelling phase: unpaired t-test for comparison between LRRC8/A siRNA-treated and scramble siRNA-treated cells under the same experimental conditions for each cell line (ns, $p>0.05$); one-way ANOVA and Neuman-Keuls multiple comparison test for comparison between different conditions of the same cell line (ns for WT, $p>0.05$; $p<0.0001$ for TRPV4, detailed statistically significant differences are shown in Table S10) and different cell lines under the same condition ($p<0.0001$, detailed statistically significant differences are shown in Table S11). For the percentage of cell volume recovery: unpaired t-test for comparison between LRRC8/A siRNA-treated and scramble siRNA-treated cells under the same experimental conditions (**$p<0.01$; ***$p<0.001$). (For WT with scramble: Ctrl n=18; 4αPDD n=20. For WT with LRRC8/A siRNA: Ctrl n=8; 4αPDD n=12. For TRPV4 with scramble: Ctrl n=12; 4αPDD n=16. For TRPV4 with LRRC8/A siRNA: Ctrl, n=13; 4αPDD, n=14).

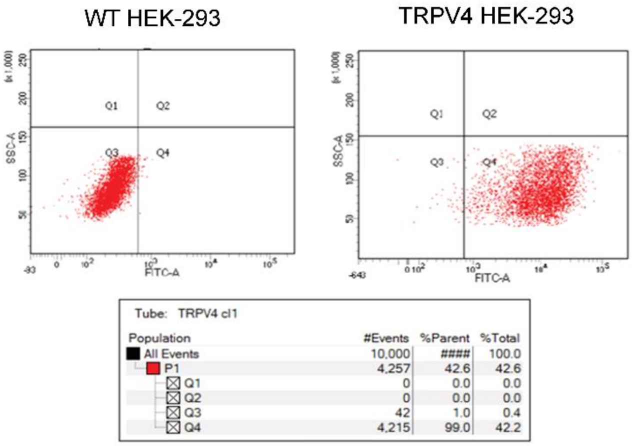

Supplementary Figure 3. Dot plots of flow cytometry on HEK-293 cells overexpressing TRPV4-EFGP (*BD FACSAria III*) and lower table showing that the 99.0% of the cells are positive for green fluorescence. Control cells are represented by WT HEK-293 cells.

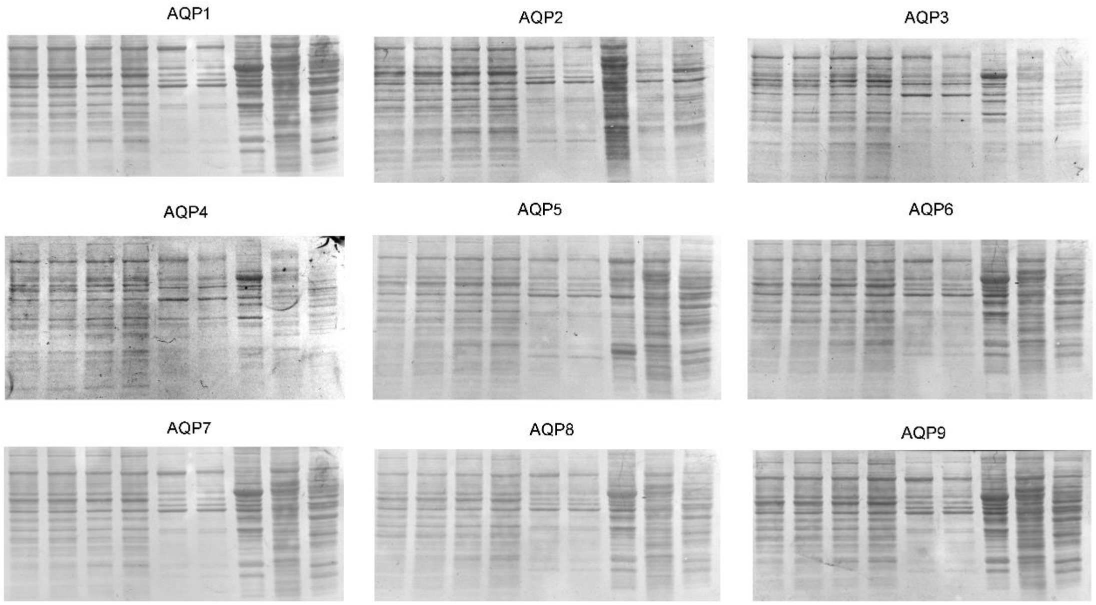

Supplementary Figure 4. Coomassie Blue staining as loading control for Western Blot data revealed for AQPs shown in Figure 7.

2 Supplementary Methods

Short-Interference RNA (siRNA). Transient transfection of siRNAs was carried out as described in the manuscript. For VRAC silencing, a SMARTpool of LRRC8/A-VRAC subunit (CCAAUAGGAUCGAGGCGCU), (CGUUUGAGUCGAUCCGAGA), (GCGCAGAGAACAACCGCUA, (GGUACAAACCACAUCGCCUA) siRNA was used. Scramble siRNA (CGAUGGAGAAGGCCAACUAGGGACU) was used as Ctrl siRNA (Dharmacon Research, Inc. (Lafayette, CO)).

Antibodies. Rabbit anti-LRRC8/A (Twin Helix, diluted 1:4000 for Western Blot; 1:400 for immunofluorescence) (Formaggio et al., 2019) antibody was used.

Sample preparation for FACS. Transfected cells were washed three times with Phosphate Saline Buffer (PBS), without calcium and magnesium, and harvested with trypsin. The number of cells per milliliter was estimated using a Bürker chamber and 1x106 cells were resuspended in filtered phosphate saline buffer added with 1 mM EDTA, 25 mM HEPES, 1% FBS. To obtain a single cell suspension, large debris and cell clusters were removed from the cell suspension by filtration through pre-wetted 50 µm filcon filters syringe type (Falcon, BD Biosciences, San Jose, CA) into 5 ml tubes, and analyzed by flow cytometry.

Flow Cytometry and Cell Sorting

A FACS Aria III (BD Biosciences, San Jose, CA) instrument was used for flow cytometry analysis. Negative controls were represented by non-transfected cells. Protein expression was evaluated on the number of processed cells and the percentage of fluorescence of the EGFP. Cells were first gated according to their light scattering characteristics (forward scatter: FCS; side scatter: SSC), and then analyzed to detect TRPV4 expression. Flow cytometry analysis was conducted with FACS Diva 8.0 software (BD Biosciences, San Jose, CA). The cells resulting positive to EGFP fluorescence were subjected to single cell sorting and collected in 96-wells plate in 10% FBS-DMEM. Cells were maintained at 37°C in a 5% CO2 incubator and then exposed to 0,8 mg/ml geneticin (http:// www.thermoscientific.com) for antibiotic resistance selection. The selected cells were maintained in culture and then analyzed by flow cytometry to evaluate TRPV4-EGFP expression.

Percentage of RVD

The percentage of volume recovery was calculated by using the following formula:

(Eq. 1):

$$\%\, RVD = \frac{(Ymax - Yo)}{(Ymax - Yrec)} \times 100$$

Where Y_{max} is the maximum fluorescence value reached after the osmotic shock, Y_0 is the baseline fluorescence value, and Y_{rec} is the fluorescence value reached after regulatory volume response.
